# Effect of Organic Loading Rate on Anaerobic Digestion Performance of Mesophilic (UASB) Reactor Using Cattle Slaughterhouse Wastewater as Substrate

**DOI:** 10.3390/ijerph15102220

**Published:** 2018-10-11

**Authors:** Mohammed Ali Musa, Syazwani Idrus, Che Man Hasfalina, Nik Norsyahariati Nik Daud

**Affiliations:** 1 Department of Civil Engineering, Faculty of Engineering, University Putra Malaysia, Serdang 43400, Selangor, Malaysia; alisulezee@gmail.com (M.A.M.); niknor@upm.edu.my (N.N.N.D.); 2 Department of Civil and Water Resources Engineering, University of Maiduguri, P.M.B., Maiduguri 1069, Borno State, Nigeria; 3 Department of Biological and Agricultural Engineering, Faculty of Engineering, University Putra Malaysia, Serdang 43400, Selangor, Malaysia; hasfalina@upm.edu.my

**Keywords:** UASB reactor, cattle slaughterhouse wastewater, biogas production, methane yield, COD removal

## Abstract

In this study, the performance of a laboratory scale upflow anaerobic sludge blanket (UASB) reactor operating at mesophilic temperature (35 °C) was examined. Cattle slaughterhouse wastewater (CSWW) was used as the main substrate. The total and effective volumes of the reactor were 8 L and 6 L, respectively. Twelve different organic loading rates (OLR) were applied and the performance was evaluated. The chemical oxygen demand (COD) removal efficiency was more than 90% during batch study. In the continuous study, COD removal was also approximately 90% at OLR 0.4 g/L d^−1^ which subsequently dropped to below 50% when the loading rate increased to 15 g/L d^−1^. Approximately 5 L/d of biogas was obtained with high methane concentration at stages VI and XI corresponding to OLR of 2 and 10 g/L d^−1^, respectively. It was observed that the concentration of volatile fatty acids was low and that the alkalinity of the wastewater was sufficient to avoid acidification. Specific methane yields of 0.36 and 0.38 LCH_4_/g COD added were achieved at OLR 7 and 10 g/L d^−1^. A hydraulic retention time (HRT) of 1 day was sufficient to remove greater than 70% of COD which correspond to 89% methane concentration. Parameters like soluble COD, NH_3_-N, pH, alkalinity, total suspended solid (TSS), fats, oil, and grease were also investigated. The results show that the UASB reactor could serve as a good alternative for anaerobic treatment of CSWW and methane production.

## 1. Introduction

Recently, the use of biodegradable waste materials to generate energy in the form of biogas has gained more popularity due to climate change [1,2]. Also, laws and regulations imposed on industrial activities regarding effluent quality standard discharge limits are now more stringent than ever before. Cattle slaughterhouses are among the many food industries that consume a considerable amount of freshwater and generate large volumes of wastewater rich in organic contaminants and nutrients [3]. The water produced during and after the operation is considered as high-strength wastewater, because of the presence of protein, fats, oil, and grease (FOG) with a high tendency for rapid acidification. The chemical oxygen demand (COD) and biological oxygen demand (BOD) are normally found to be tens of thousands mg/L [4]. This range varies from 18,000 mg/L to 43,000 mg/L [5], but the strength differs from one industry to another depending on the number of animals slaughtered. The chemicals and detergents used for cleaning of the abattoir facilities also influence the concentration of COD. The presence of FOGs in CSWW is recognized as one major component that largely contributes to the BOD and COD which portrays waste as a potential source of biogas production [6]. Also, its recalcitrant nature could result in numerous problems such as foul odor, clogging of pipes, bacterial cell surface adhesion, and scum layer formation. These challenges could affect smooth biogas transition within the reactor [7].

Discharge of wastewater without proper treatment constitutes an environmental problem to both flora and fauna. The United State Environmental Protection Agency (US EPA, 2004) [8] has classified slaughterhouse wastewater as one of the most harmful waste substances to be released to the environment. Furthermore, untreated discharge of wastewater to the receiving water body could inhibit light penetration to aquatic animals and encourage eutrophication as well as unpleasant aesthetic value [9]. Table 1 presents the prominent characteristics of meat processing wastewater effluent and its limits from various jurisdictions worldwide.

Conventional treatment of slaughterhouse wastewater include physicochemical (primary) which is the separation of floating and settleable solids typically by sedimentation or coagulation/flocculation. Secondly, the biological treatment (secondary) is system where lagoons and activated sludge are commonly utilized. Lastly, the advanced oxidation ditches (tertiary) are systems that require high aeration (oxygen) to remove nitrogen (N_2_) and phosphorus (P) [11,12]. These methods are used to produce excellent effluent quality; however, the general drawbacks include large space requirement, high cost of aeration, huge volume of sludge generation with offensive odor, and capital intensive [13]. Moreover, they lack focus on energy recovery, instead these processes form part of the pathways where potential greenhouse gases such as methane (CH_4_) and carbon dioxide (CO_2_) escape to the atmosphere [14].

Therefore, a reliable, cost-effective and efficient treatment process is required to remove wide range of pollutants and recover energy. Water ready for discharge or reuse into the environment should not only be free from organic or mineral contaminants, but should also take into consideration the presence of bacteria, pathogens, and viruses. Otherwise, the standards set by the various environmental regulatory bodies could be violated. In an attempt to meet the stringent water quality regulations, several techniques including physical, chemical, and biological methods of wastewater treatments have been used over time. But, the biological method through the anaerobic digestion process appears to be a promising technique owing to less sludge production, better energy recovery and the overall low cost of application [13,14,15]. Basically, there are four diverse groups of microorganism responsible for the degradation process. All of the four metabolic reaction stages usually occur in the absence of oxygen. These include hydrolysis, acidogenesis, acetogenesis, and methanogenisis [16]. Besides biogas production, other byproducts like liquid or solid residue are also produced, which are applied to the soil as fertilizer or amendment [17]. Under strict anaerobic condition, a typical biogas consists of methane (CH_4_) and carbon dioxide (CO_2_). It may also contain significant amount of undesirable traces of compounds like hydrogen sulfide (H_2_S) and ammonia (NH_3_) [18]. Most of the organic fraction of slaughterhouse contained large amount of lipids that consist of triglycerides and long chain fatty acids (LCFA). However, compounds such as triglyceride easily hydrolyze to form LCFA and glycerol. Inhibition of the anaerobic digestion process may be due to high accumulation of LCFA. It could affect the normal function of acetogenic and methanogenic bacteria as a result of its toxicity [19]. Moreover, adsorption of the surface active acids on to the cell wall may also be part of the mechanisms responsible for anaerobic digestion inhibition during transportation and protection processes [20].

Wastewater produced from meat processing activities is highly concentrated in terms of COD, BOD, and FOG. Also, treatment of CSWW can be as energy intensive as municipal wastewater [21]. Therefore, the goal of this work is to evaluate the efficiency of an upflow anaerobic sludge blanket reactor for treatment of CSWW and biogas production at different organic loading rates.

## 2. Materials and Methods

### 2.1. Substrate

The cattle slaughterhouse wastewater sample was collected directly from the discharge point of the abattoir in Selangor, Malaysia. This slaughter house is the biggest in the state of Selangor. Specimens were collected in three containers, each of 25 L volume. They were immediately transported to a public health laboratory situated at the Faculty of Engineering, Universiti Putra Malaysia. Prior to use, the content of the three containers were mixed homogeneously and screened to remove particles larger than 5 mm. Tests such as pH, COD, BOD_5_, and ammonia nitrogen were immediately carried out after mixing. The sample was then stored in a refrigerator at 4 °C for further use. Table 2 shows the characteristics of the sample used for the experiment.

### 2.2. Inoculum

The seed sludge used in the UASB reactor was collected from the Faculty of Engineering wastewater treatment plant at Universiti Putra Malaysia (UPM). It contained a black mixture of fine particles with relatively small size granules. After three replicates, the total chemical oxygen demand (COD), soluble chemical oxygen demand (_S_COD), total suspended solids (TSS), and volatile suspended solid (VSS) achieved were 0.24, 0.158, 11.24, and 9.87 g/L, respectively. These parameters were determined based on the procedures given in the standard methods for the examination of water and wastewater of the American Public Health Association Manual [22].

### 2.3. Analytical Methods

The performance evaluation of the UASB reactor was carried out regularly at an interval of 2 days. Parameters such as total COD, alkalinity, total solid (TS), total, FOG, and volatile suspended solid (VSS) were determined using standard methods for the examination of water and wastewater by the American Public Health Association (APHA) [22]. Other parameters such as TSS and SCOD were carried out after filtration using a 0.47 μm filter on the vacuum pump. Other parameters like ammonia nitrogen (NH_3_-N), color, and turbidity concentrations were measured using multiparameter portable calorimeter HACH system (DR 900). The methods used for each of these parameters were; Spectrophotometer (Salicylate powder pillow method 8155) for NH_3_-N, Platinum-Cobalt Standard Method 8025 for color, while the Absorptometric method 8237 was used for the turbidity measurement. A pH meter (PHS-3C, INESA, Shanghai, China) was used to determine the pH of the system. The volume of biogas produced was measured by the water displacement method. In addition, the biogas composition analyses were examined using a gas chromatograph (HP 6890 N) (Agilent, Santa Clara, CA 95051, USA) equipped with a thermal conductivity detector (TCD) (Agilent, Santa Clara, CA 95051, USA). The column was HP Molesieve (Agilent Technologies, Santa Clara, CA, USA) of 30 m length × 0.5 mm ID × 40 μm film thickness capillary column. The split less inlet, oven, and TCD detector temperatures were all kept at 60 °C, 70 °C, and 200 °C, respectively. Argon functioned as the carrier gas while nitrogen was used as the makeup gas. The peaks of samples were identified and the concentration was calculated based on the retention time and peak area of known standards (Sigma Chemical, St. Louis, MO, USA).

### 2.4. Biochemical Methane Potential Test (BMP)

In order to determine the maximum volume of methane that can be produced from a specific substrate a biochemical methane potential test was performed following the procedure reported by Angelidaki et al. [23]. However, BMP may also be used to estimate the fraction of the substrate that can be converted to methane. The tests were carried out in (duplicates) to achieve reliable results. Two scotch bottles of 1000 mL containing 450 mL inoculum (sludge) and substrate of equal volume of inoculum were filled up to 900 mL, leaving 100 mL for biogas head space. The substrate biomass to inoculum ratio was kept at ratio of 1:1. Additionally, two blank bottles containing 450 mL inoculum topped up to 900 mL with tap water were used as controls. All bottles were flushed with 100% nitrogen gas (N_2_) for 1 min to avoid the presence of air in the head space before closing with the rubber stopper. The digester was then incubated in a water bath at 35 °C with the water level at the bottle neck. Each digester was shaken every day for 1 min in the water bath until the biogas production cease after eleven (11) days. The biogas production was observed by the water displacement method using a graduated measuring cylinder. The methane content was analyzed by gas chromatography and calculated by subtracting the corresponding values for control and substrate runs. Other physicochemical parameters such COD, TSS, VSS, and NH3-N were also examined before and after the BMP test.

### 2.5. Experimental Process Flow

**Figure 1:**
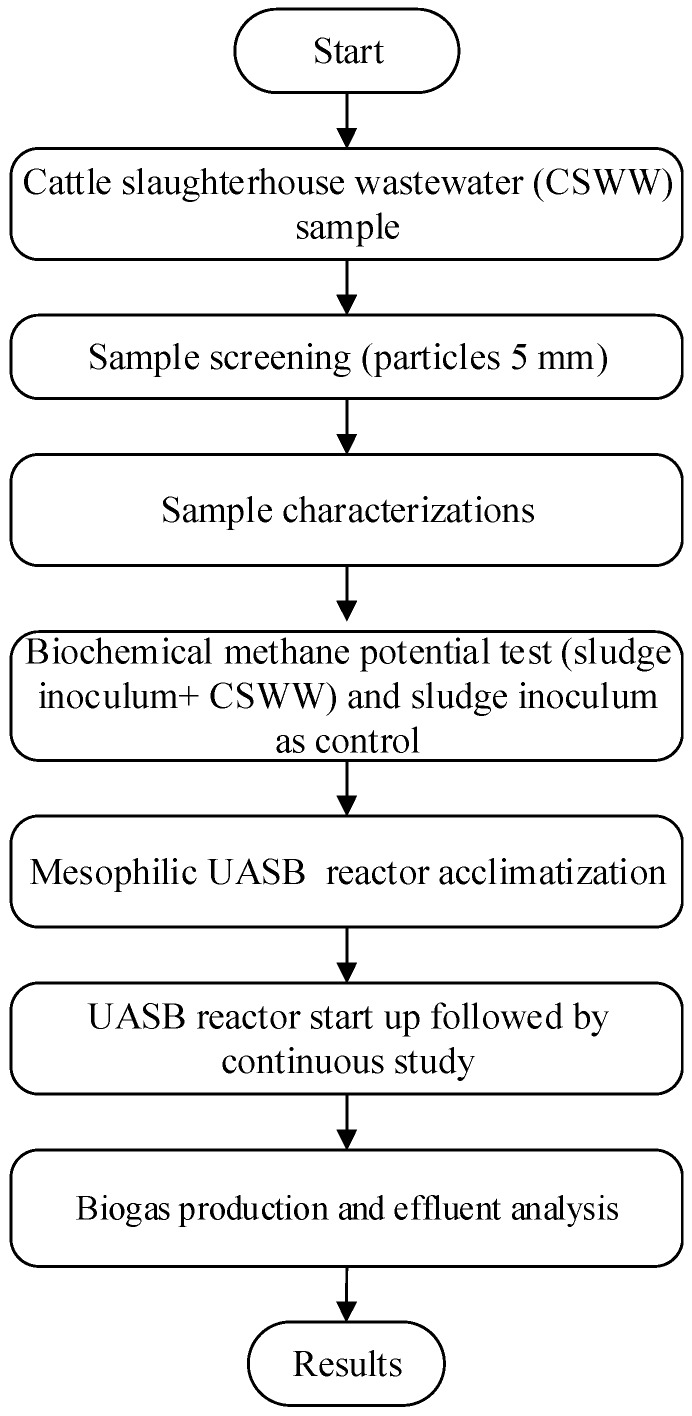
Flowchart of the experimental phases that was used in the study.

Figure 1 shows the process flowchart of the experimental procedure of the anaerobic digestion of CSWW conducted following steps described in Guimarães et al. [24]. An effluent characteristic for both batch and continuous studies was evaluated in triplicates and average values were considered.

### 2.6. UASB Reactor Set-Up

**Figure 2:**
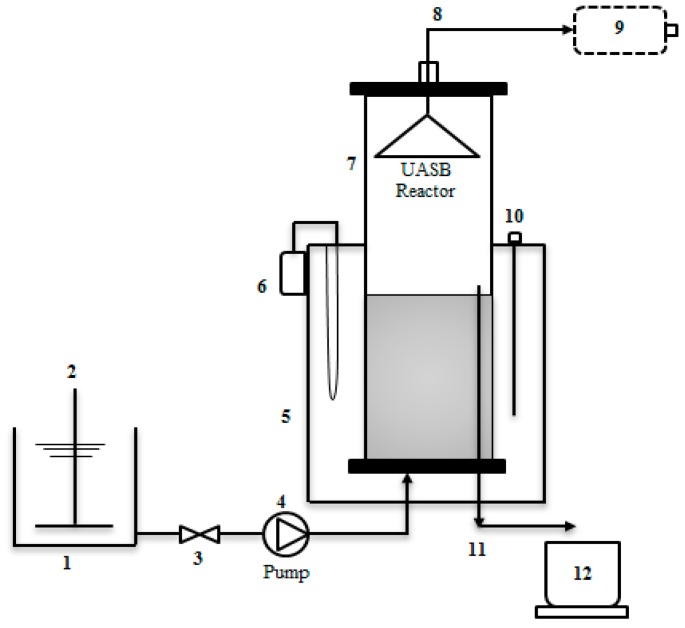
Schematic diagram of upflow anaerobic sludge blanket (UASB) reactor used in the study. Legend: 1—Influent; 2—Stirrer; 3—open and close Valve; 4—Peristaltic pump; 5—Water bath; 6—Heating element; 7—PVC column reactor; 8—Biogas outlet; 9—Tedlar bag; 10—Thermometer; 11—Effluent outlet; 12—Collection tank.

Figure 2 shows the laboratory scale of a 15 cm diameter and 60 cm height PVC column UASB reactor setup with total and effective volumes of 8 L and 6 L, respectively. The effective volume occupies 75% of the volume from the bottom. The sludge granular formations at the bottom have better settling properties and do not wash out easily. In addition, this allows for a higher OLR potential of the reactor. The formation of well-stabilized granular properties for a long period of time enhances the biomass retention within the reactor [25]. Feeding of the reactor was done from the bottom of the system through a blanket of biologically activated sludge, which is largely in the form of granular aggregates. This was done to provide sufficient contact between the CSWW (influent) and sludge blanket-containing microbes. The upflow velocity exhibits less effect on the movement of microbes, as the feeding was done at an interval of time over 24 h. This strategy would drastically increase biogas flow and reduce the chances of scum formation that usually prevents the steady flow of biogas out of the reactor.

The reactor start-up commenced by acclimatizing the environment (UASB reactor) using synthetic wastewater with a composition as shown in Table 3. The procedure follows a synthetic wastewater prepared according to Idrus et al. [26]. The COD concentration was 203,000 mg/L and the acclimatization period lasted for three weeks. The plant where inoculum was collected operates under mesophilic condition and as such, the temperature shift was needless. To avoid any change of state of microbial functions, the UASB reactor was also maintained at 35 °C by heating the water bath jacket throughout the experiment. However, the reactor was purged with 100% N2 gas prior to startup. Table 4 depicts the summary of the strategy employed during the UASB reactor startup. Acclimatization of the environment was immediately followed with Batch study (same as in the case of BMP test) using raw slaughterhouse wastewater at an organic loading rate of 0.2 g/L d^−1^. The raw CSWW was fed once until biogas production ceased on the eleventh day (HRT = 11 day). During the batch study, the reactor performance had greatly improved in terms of total COD and soluble COD removal. On the twelve day, the continuous study (Run II) was achieved by daily effluent discharge followed by feeding the same amount of CSWW using a peristaltic pump at a flow rate of 0.6 L/m, as shown in Figure 2. Exclusive feeding of the CSWW was achieved by increasing OLR after each run. HRT of day 1 was also maintained by reducing the dilution factor of the substrate until the reactor show signs of decreased performance. The loading rates were 0.4, 0.7,1, 1.5, 2, 2.5, 3, 5, 7, 10, and 15 g/L d^−1^.

Previous research on anaerobic digestion revealed that, during reactor startup, an appreciable level of biomass degradation could be achieved by increasing influent substrate concentration at constant HRT [27]. For instance, the studies of Najafpour et al. [28] and Zhang et al. [29] have shown that high COD removal efficiency can be achieved by increasing organic loading rate and maintaining constant HRT in the treatment of substrate with high lipids. Furthermore, Ghaniyari et al. [30] and Sowmeyan et al. [31] revealed that anaerobic degradation of wastewater in a bioreactor could startup by increasing OLR and reducing HRT without changing influent COD concentration. Therefore, the reactor in this work was maintained at constant HRT throughout the experimental period. Also, the pH of the system was closely monitored on daily basis to avoid pH drop to below 6.5 which could cause system failure.

### 2.7. Mesophilic UASB Reactor Performance Study on CSWW

The performance of the UASB reactor in the treatment of CSWW was first investigated in batch mode. The organic loading rate, OLR, and hydraulic retention time, HRT, remained unchanged. To assess the performance treatment of the system, HRT, feed, and biomass concentration were all constant. Subsequently, the effect of increasing loading rate on the treatment efficiency was evaluated by varying the feed COD concentration whilst operating at a constant HRT. The influent substrate, effluent discharge flow rates, biogas production, and pH where observed on daily basis.

### 2.8. Scanning Electron Microscopy Analysis

Surface morphology of the sludge (inoculum) before and after contact with CSWW was conducted using a scanning electron microscope (S-3400N SEM HITACHI, Milpitas, CA, USA). The samples were initially coated with gold to induce conductivity. The surfaces of the samples were viewed at 300× magnification with the objective of obtaining some information on the behavior of the materials.

## 3. Results and Discussion

### 3.1. Biochemical Methane Potential of CSWW

**Figure 3:**
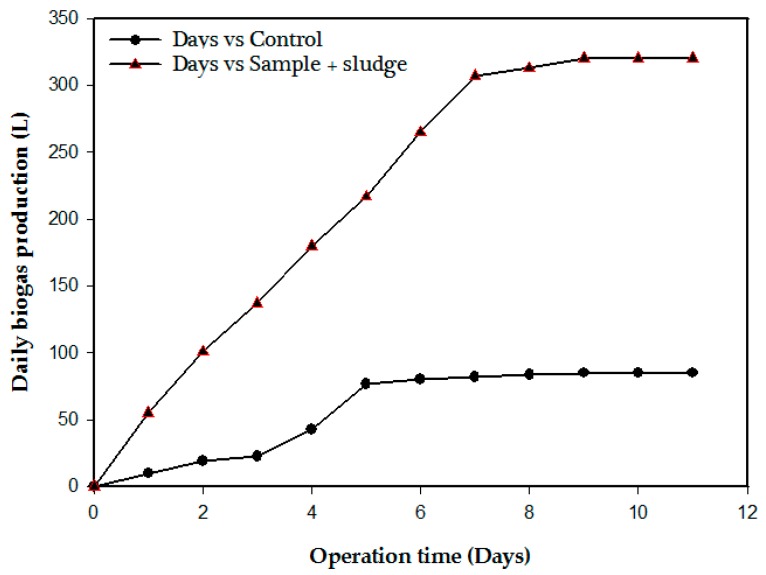
Result of biochemical methane potential tests.

Figure 3 shows the biochemical methane potential test carried out to determine the degradability of CSWW. The OLR of the feed introduced to the scotch bottles was 0.2 g/L which correspond to 0.4 g/L COD. An average total cumulative biogas produced in the mixture of feed to inoculum (F/I) ratio was 320 mL and 85 mL in the blank bottles. The composition of the biogas in the (feed/inoculum) ratio was 0.80% oxygen, 18.45% nitrogen, 75.80% methane, and 4.92% carbon dioxide. After deducting the amount of biogas produced in the average of blank bottles, the resulting biogas produced by the feed was 235 mL. Based on the percentage of methane gas present in the biogas (75.80%), the methane yield corresponds to 178 mL. Therefore, 0.18 gCOD/L is the actual amount that produced the 178 mL of methane. Hence, the methane produced by the CSWW used as feed was 31 mL CH_4_/gCODadded. The COD, SCOD, and TSS removal efficiencies were 95%, 98%, and 98.5%, respectively. Ammonia nitrogen concentration (NH_3_-N) and alkalinity ratio (IA/PA) were 38 mg/L and 0.2, respectively.

### 3.2. Effect of Organic Loading Rate on COD and SCOD Removal Efficiency

**Figure 4:**
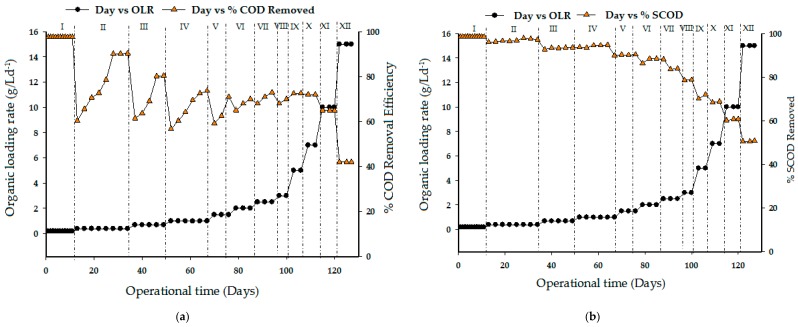
Effect of organic loading rate on (**a**) chemical oxygen demand (COD) removal efficiency and (**b**) soluble COD removal.

Figure 4 shows the changes in OLR corresponding to COD removal efficiency in the UASB reactor during the period of the operation. In the beginning, 0.2 g/L d^−1^ was fed to the reactor for the batch studies (Run I). It took eleven days for the reactor to completely stabilize the biomass. The HRT was observed to take too long due to the fluctuation in daily biogas production. Also, it was observed that at the lowest organic loading rate of more than 98% COD removal was realized. After batch feeding attained a stable state, the reactor was then fed continuously. Prior to subjecting the reactor to higher OLR, continuous study was start-up with 0.4 g/L d^−1^ (Run II). During this period, biodegradation took a longer period before final stabilization. It was observed that the COD removal efficiency was 90% for three consecutive days. However, the period the reactor took before stabilizing was probably due to the shock received during the change of state of the OLR. The influent COD was further increased to 0.7 g/L d^−1^, which corresponds to an organic loading rate of 1.4 g/L^−1^. A continuous increase in OLR with fat rich wastewater was maintained until the COD removal efficiency declined to 42% at 15 g/L d^−1^. The soluble COD removal efficiency of the UASB reactor, in terms of OLR, is shown in Figure 3b. At the initial state of the study, the loading rate was maintained at 0.2 g/L d^−1^ and subsequently increased from 0.4 to 15 g/L d^−1^ during continuous studies. SCOD removal efficiencies showed an increasing trend from a maximum of 92% at 1.5 g/L d^−1^ OLR to approximately 80 to 70% between run VI and IX. However, at the beginning of each phase where OLR increased, there existed a corresponding decrease in removal efficiency; however, the system recovered shortly and adapted to the new condition with time. It can be observe that at an OLR of 7 g/L d^−1^, the COD and SCOD removal were approximately 70% and 85% and this was regarded as the upper limit for satisfactory performance of this type of wastewater under the conditions of this study.

Comparatively, the results obtained in this work showed a better performance compared to the values obtained in a past paper [32]. The COD and SCOD removal efficiencies were (97% ± 2%) and (95% ± 3%) at OLR 4.5 kg TCOD m^−3^ d^−1^. This is because a large amount of energy was expended during the aeration process and the membrane technology was further applied to achieve better efficiency in the treatment of wastewater. Furthermore, the concentration of raw COD in this study was much higher compared to the raw COD of 2711 ± 487 achieved in the aforementioned study. Similar results of COD and SCOD removals were also obtained in the research of Rajakumar et al. [33], but the data showed lower performance of this study, especially when HRT, OLR, and initial COD concentrations of the two substrates were compared. Moreover, these concepts have been positively reinforced by the findings of Nachev et al. [34] which presented high COD removal efficiency.

### 3.3. Biogas Production, Percentage of Methane Yield, and Specific Methane Production

**Figure 5:**
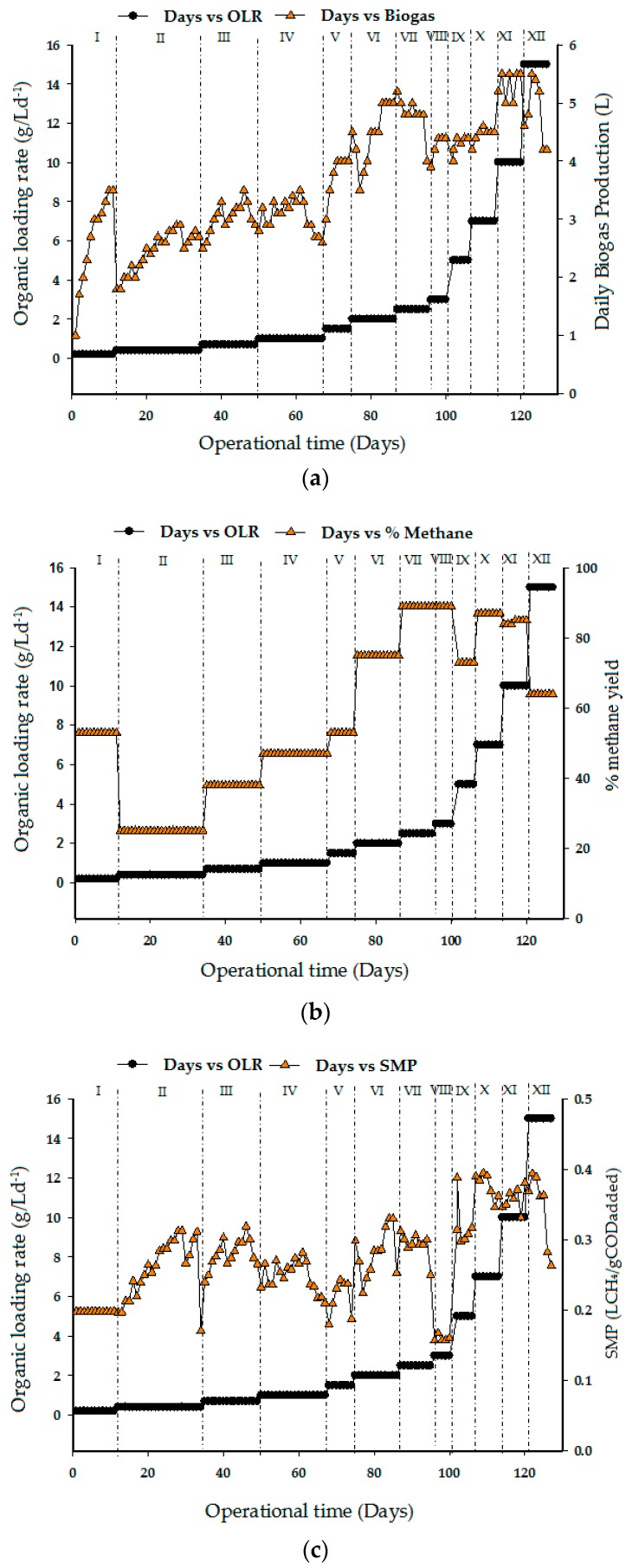
Effect of organic loading rate on (**a**) biogas production, (**b**) percentage of methane yield, and (**c**) specific methane production.

Figure 5a reveals the total amount of biogas produced during the period of UASB reactor operation. After acclimatization and subsequent batch studies that lasted for 11 days, the initial OLR 0.4 g/L d^−1^ was gradually elevated to 15 g/L d^−1^ until biogas production reduced to 4.2 L in the last phase. Biogas production increased tremendously at OLR 2.5 and 10 g/L d^−1^, which corresponds to 4 and 5.5 L biogas, respectively. The increasing OLR by feeding the CSWW in run VII and XI with COD concentrations of 5 and 20 g/L d^−1^, respectively, yields methane concentrations of 89% and 85%, as shown in Figure 5b. Subsequently the OLR was then increased to 15 g/L d^−1^ in (run XII), after which the COD and methane concentration in run XI remained at 64%. It is clear that a decrease in methane concentration of biogas decreases with the increase in OLR at some point. However, the specific methane yield is still higher within these phases as shown in Figure 5c. A slight decrease of methane yield was experience in run VIII. This was attributed to the shock received by the system due to changes in the OLR that affect the methanogenic activities. Therefore, counter measures are required to avoid continuity of the process. From the initial phase (run I), variation in CH_4_ content was observed from a minimum of 0.1981 LCH_4_/gCOD added (day 1) to a maximum of 0.3950 LCH_4_/gCOD added at OLR 7 g/L d^−1^. A similar trend was recorded in runs XI and XII until the specific methane remained quite constant and close to the average value of 0.2631 LCH_4_/gCOD added.

In general, Figure 5a–c depicts the biogas production, percentage of methane, and the specific CH_4_ production rates with respect to the increasing OLR values. Conversely, the biogas and the CH_4_ production rates decreased markedly with increasing COD loading rates in the last phase (Run XII). Interestingly, when the UASB system was operated at COD loading rates below 3 g/L d^−1^, the process performance in terms of COD removal efficiency and biogas production rates was observed to be higher, because the system was under a low range of organic loads. The trend in Figure 5a,b proved that an increased COD loading rate causes an increased level of organic substrates that are available for conversion to biogas resulting in CH_4_ production. However, at some points of the trend of biogas and methane production rates, an increase in organic loading rate also caused a reduction in the biogas and CH_4_ production rates. But most importantly, no increases in toxicity or volatile fatty acid (VFA) accumulation in the reactor were noticed. Hence, the decrease in the overall yield at 15 g/L d^−1^ OLR could be due low hydraulic retention time for microbe to consume the substrate.

The investigation of Marcos et al. [35] also supported the findings of the present work. However, they reported a biogas production yield of 18 L after mixing with serum slaughterhouse waste and approximately 10 L of biogas for purely slaughterhouse waste on daily productions. But, their system used a continuous stirrer tank reactor (CSTR) where mixing was provided with the use of an electric motor that led to sufficient contact between the microbes and the biomass. A comparison of these results with others investigations also obtained from UASB reactor working under similar conditions reveal quite a satisfactory result. For instance, Kwarciak-Kozłowska et al. [36] revealed maximum COD and BOD_5_ removal efficiencies of 85% and 82%, respectively, at HRT of 6 days. The removal of organic pollutants decreased with the shortened HRT and the daily biogas production decreased with an increase in the HRT. The fermentation process of the wastewater was highly characterized by high methane content (75%). However, from an economic point of view, HRT is too long compared to the present study.

### 3.4. Effect of Alkalinity, pH, and Ammonia Nitrogen

The high protein and lipid content of slaughterhouse wastewater makes it a challenging material for anaerobic digestion due to the inhibition caused during degradation. Functional anaerobic digestion system depends on the buffering capacity and the degree of adaptation of the microorganisms [37]. Alkalinity is an important parameter in anaerobic digestion that shows the capability of a solution to withstand a drop in pH produced by the release of organic acids. It is also termed as the system buffer capacity [38]. The ability of methanogenic bacteria to resist higher VFA accumulation is highly dependent on the alkalinity value of the system as well its buffering capacity. Thus, methane production processes in a bioreactor are basically a function of alkalinity and pH stability [39]. In anaerobic digestion, microorganisms have a working range of pH. Methanogens are sensitive to pH between 6.5 and 7.5 and have optimal pH of between 7.0 and 7.2 [40]. For efficient methane production, the anaerobic digestion process normally operates at the aforementioned optimal pH range. But, the formation of degradable intermediates (VFA) tends to lower the pH of the bioreactor during operation. Changes in pH of the bioreactor during operation are easily detected through alkalinity (gCaCO_3_/L) which is the main indicator of buffering capacity of the system. According to Mata-Alvarez et al. [41], desirable alkalinity in bioreactor is between 2000 and 4000 mg CaCO_3_/L and the VFA/alkalinity ratio should be less than 0.3.

**Figure 6:**
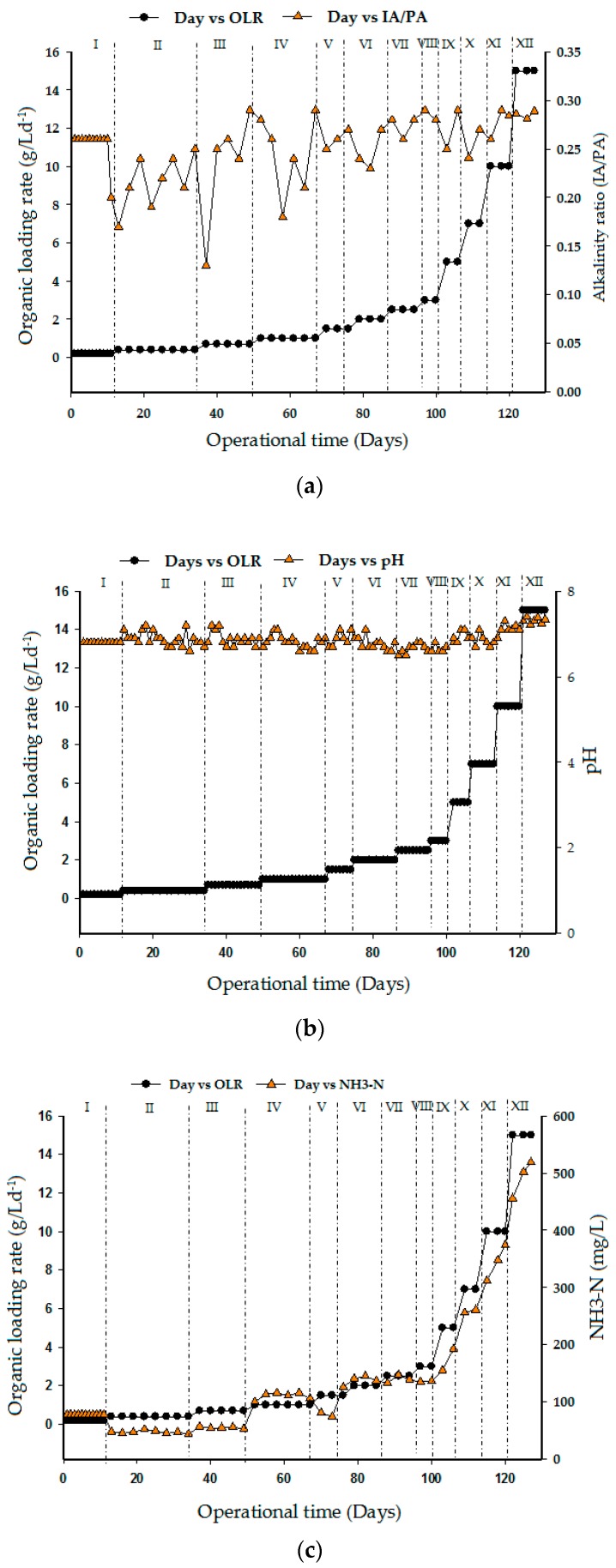
Effect of organic loading rate on (**a**) alkalinity ratio, (**b**) pH, and (**c**) ammonia/nitrogen.

Figure 6a–c shows the alkalinity, pH, and ammonia nitrogen profiles at various OLR loading rates. The maximum alkalinity ratio attained throughout the period of operation was 0.29 which depicts a stable operating performance. Likewise, the VFA concentration of the effluent was less than 0.60 g/L with no major fluctuations detected from other parameters (pH and CH_4_ concentration). Furthermore, the pH values were consistent with the increased levels of OLR biogas production and COD loading rates as shown in Figure 6b. Hence, the effluent from this reactor exhibited high buffering capacity and, as such, does not require pH correction. In addition, ammonia/nitrogen was measured at one day intervals during fermentation and methanation phases shown in Figure 6c. The ammonia nitrogen NH_3_-N level in the effluent was observed to keep increasing in values corresponding to the increase in COD levels and organic loading rates. Interestingly, the level of ammonia increase in the effluent at 35 °C neither inhibits nor caused any significant changes in the reactor operation. It is evident, that process instability due to ammonia often results in the accumulation of volatile fatty acids (VFAs). Again, this leads to a decrease in pH and thereby reduces the concentration of fatty acids. The process was carried out without inhibition even when the concentration of NH_3_-N increased from 78 mg/L to a value above 500 mg/L. The absence of inhibition in the system may be attributed to the higher activity of mesophiles, which were primarily responsible for converting the proteins to CH_4_ gas during the fermentation process. The quantity of ammonia generated from an anaerobic biodegradation of organic substrate can be estimated using stoichiometric [42].

CaHbOcNd + ((4a − b − 2c + 3d)/4) H_2_O → ((4a + b − 2c − 3d)/8) CH_4_ + ((4a − b + 2c + 3d)/8) CO_2_ + dNH_3_ (1)

In order to counteract the effect of high ammonia nitrogen accumulation in the anaerobic digestion process frequent maintenance, a change in the intracellular pH of methanogens, and the inhibition of specific enzyme reactions are some of the numerous suggested pathways for overcoming ammonia inhibition.

### 3.5. Total Suspended Solid and Oil and Grease Removal Profile

**Figure 7:**
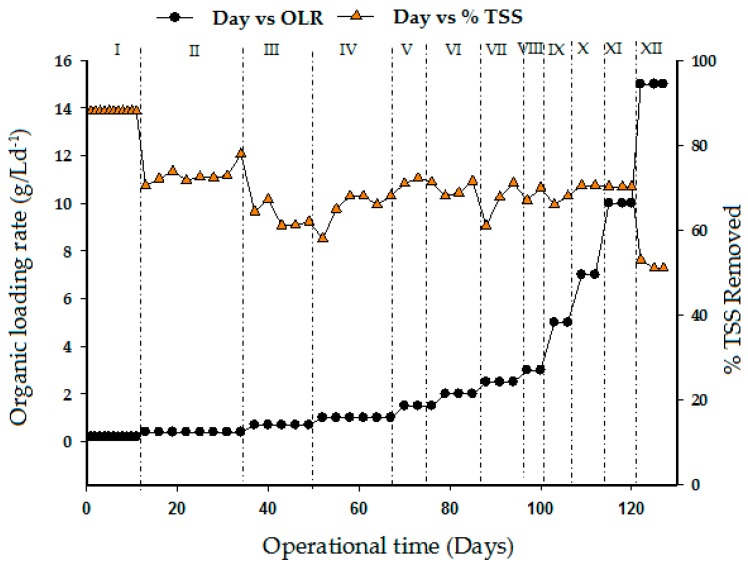
Effect of organic loading rate on total suspended solid removal efficiency.

The TSS concentration in the effluent was measured for the purpose of knowing the insoluble organic present in the treated cattle slaughterhouse wastewater. TSS was also used in this study to measure the performance of the UASB reactor at an HRT of 1 day with different organic loading rates. As a record of performance, Figure 7 shows the TSS concentration in the effluent of treated CSWW after COD removal. The raw substrate TSS concentration of 1955 mg/L was reduced to 47–158 mg/L after treatment at OLR 15 g/L d^−1^. The TSS removal during the continuous study was between 85 and 72%. The minimum TSS removal percentage was 72%, obtained at OLR 10 g/L d^−1^ and HRT of 24 h. A sudden decrease in TSS removal efficiency to 50% was observed in stage XII. However, the TSS removal efficiency of the UASB reactor does not only depend on the anaerobic digestion process, but also on the physicochemical reactions within the effluent biomass which in turn facilitates its detainment within the reactor. This phenomenon is similar to experiment reported in a previous study by Tawfik et al. [43]. In their studies, the UASB reactor was operated at a constant HRT of 24 h throughout the study, while OLRs varied from 1.9 to 4.4 kgCOD total/m^3^.d due to changes in influent composition. The results clearly demonstrate that the UASB reactor achieved substantial reduction of TSS removal of 72%. But, the treatment performance was achieved by applying the activated sludge (AS) system coupled with the UASB reactor.

**Figure 8:**
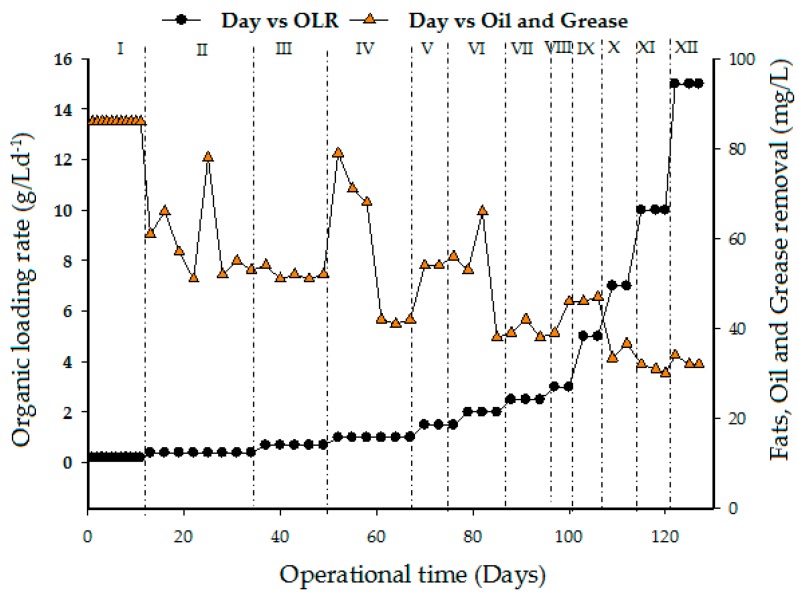
Effect of organic loading rate on fats, oil, and grease removal.

Fats, oil, and grease (FOG) mainly consists of long chain fatty acids (LCFA) that are bonded to glycerol, esters, waxes, phospholipids, sterols, and sterol esters [44]. Figure 8 demonstrates the performance of the UASB reactor in removing oil and grease. The concentration of oil and grease removal decreases with an increase in oil OLR. However, a serious decrease in removal (below 40 mg/L) was recorded in the last three phases (X, XI, and XII). Hence, anaerobically treated effluents usually require additional post-treatment to remove organic matter and other constituents like total nitrogen, phosphorus, and pathogenic organisms to acceptable limits. Based on the results obtained, the quality of the effluent did not comply with the set standard A and B of the department of environment Malaysia [45].

### 3.6. Scanning Electron Microscopy

**Figure 9:**
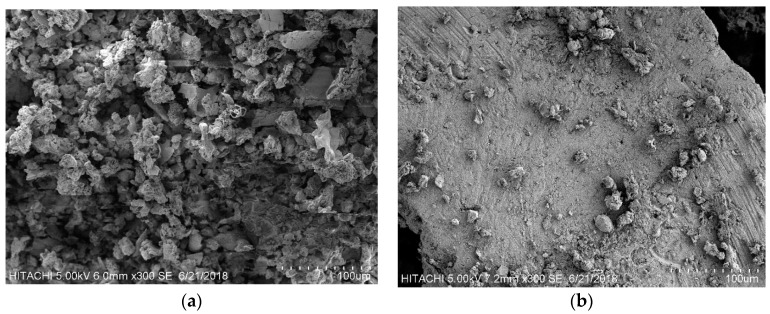
SEM image of the sludge inoculum (**a**) before contact with CSWW and (**b**) after contact with CSWW.

Scanning electron microscopy was used to describe the surface morphology of the mesophilic sludge before and after contact with CSWW as shown in Figure 9. It can be observed that after 127 days of operation, the activated sludge becomes finer with compacted granules. Thus, no sludge washes out and a large proportion of the microbial community was retained which led to high COD removal efficiency and biogas production. This finding is consistent with the result found in Wu et al. [46]. Finer granulation of sludge in UASB is one important factor that usually contributes to high COD removal efficiency [47]. However, the granulation of the sludge in this study has not significantly affect COD removal efficiency, especially in the last run (run XII) where the COD removal was less than 50%. This could be attributed to high color and turbidity and TSS of the influent substrate.

## 4. Conclusions

The organic loading rate (OLR) is an important parameter that significantly affects microbial ecology and characteristics of upflow anaerobic sludge blanket (UASB) reactor. In this work, a UASB reactor was operated at mesophilic temperature (35 °C) for the treatment of slaughterhouse wastewater. The COD and SCOD removal efficiencies were 73% and 85%, respectively, at an optimum OLR of 7 g/L d^−1^. Generally, daily biogas production increases with increasing organic loading rate. Likewise the yield coefficient of methane production also increases with the load rise, reaching 0.38 LCH_4_/gCODadded at OLR 10 g/L d^−1^ with an influent COD concentration of 6 g/L d^−1^. The maximum alkalinity ratio attained was 0.29 and this demonstrates a stable operating condition that is enough to prevent drop in pH to below 6.5. Ammonia/nitrogen serves as a nutrient for bacteria. However, its presence in high concentration could inhibit the reactor’s performance.

Therefore, the ammonia/nitrogen concentration in the reactor effluent did not affect the performance since it does not exceed the permissible limit. The influent TSS was 1955 mg/L and more than 70% was removed at 10 g/L d^−1^. It was observed that 1 day of HRT is sufficient to achieve organic matter removals. Also, the comparison with literature studies for similar wastewater indicates that this study attained higher treatment efficiencies compared with others at higher sustainable loading rates. The overall quality of the effluent from the UASB reactor did not comply with the standard (B) for water quality discharge parameters like (COD = 200 mg/L, Oil and grease = 10 mg/L, and NH_3_-N = 20 mg/L) set by the Department of Environment (DOE) Malaysia. Therefore, post-treatment of the effluent is required; if possible the use of renewable raw materials (waste from biomass) should be encouraged rather than applying chemicals or other energy demanding resources. The presence of high protein, fats, oil, and grease in the wastewater portray the waste as a strong source of energy in the slaughterhouse environment. Also, the availability of the waste is an additional advantage to scale up energy production to a commercial scale.

## Figures and Tables

**Table 1:** Comparison of standard limits for slaughterhouse wastewater discharge from different jurisdictions worldwide [10].

Parameter	World Bank	EU	USA	Canada	Colombia	China	India	Australia
BOD_5_ (mg/L)	30	25	16–26	5–30	50	20–100	30–100	5–20
COD (mg/L)	125	125	n.a.	n.a.	150	100–300	250	40
TN (mg/L)	10	10–15	4–8	1.25	10	15–20	10–50	10–20
TOC (mg/L)	n.a.	n.a.	n.a.	n.a.	n.a.	20–60	n.a.	10
TP (mg/L)	2	1–2	n.a.	1.00	n.a.	0.1–1.0	5 F	2
TSS (mg/L)	50	35–36	20–30	5–30	50	20–30	100	5–20
pH	6–9	n.a.	6–9	6–9	6–9	6–9	5.5–9	5–9
Temperature (°C change)	n.a.	n.a.	n.a.	<1 °C	n.a.	n.a.	<5 °C	<2 °C

**Table 2:** Characterization of raw cattle slaughterhouse wastewater (CSWW) used in this study.

Parameter	Unit	Average Value
BOD_5_	mg/L	6345 ± 86
COD	mg/L	14,720 ± 312
_S_COD	mg/L	767 ± 121
TS	mg/L	7800 ± 86
TSS	mg/L	1955 ± 65
TDS	mg/L	635 ± 23
VSS	mg/L	1320 ± 32
pH	mg/L	6.66 ± 1
FOG	mg/L	205.6 ± 12
NH_3_-N	mg/L	69 ± 2
TN	mg/L	780 ± 16
Color	Pt Co	500 ± 5
Turbidity	FAU	576 ± 11
Temperature	°C	27.2 ± 2
Alkalinity	mg/L as CaCO_3_	485 ± 14

**Table 3:** Synthetic wastewater composition.

Material	Quantity	Unit
Yeast (granular form)	23	g
Urea	2	g
Sugar	11.5	g
Ammonium phosphate (NH_4_)2HPO_4_	3.4	g
Full cream milk	144	mL
Raw blood from cattle slaughter point	5.75	mL
Tap water	Full to 1 L	

**Table 4:** Start-up of the UASB reactor.

Fixed Parameter			Units			Value
Temperature			°C			35 ± 1
HRT			hr.			24
pH			‒			6.5
Experimental Run	Day	Influent COD concentration (g/L)	Feed flow rate (L/d)	Maximum COD removal efficiency	OLR (g/L d^−1^)	Average effluent pH
				(%)		
Batch feed						
Stage I	1–11	0.4	3	98.6	0.2	6.7
Continuous feed						
Stage II	11–34	0.8	3	90.3	0.4	6.95
Stage III	34–49	1.4	3	80.4	0.7	6.87
Stage IV	49–67	2	3	73.6	1.0	6.63
Stage V	67–74	3	3	71.0	1.5	6.72
Stage VI	74–86	4	3	70.2	2.0	6.85
Stage VII	86–95	5	3	73.0	2.5	6.77
Stage VIII	95–100	6	3	70.0	3.0	6.60
Stage IX	100–106	10	3	72.5	5.0	6.7
Stage X	106–113	14	3	72.1	7.0	6.8
Stage XI	113–120	20	3	65.0	10.0	7.1
Stage XII	120–127	30	3	42.0	15.0	7.3

## Abbreviations

BOD	Biochemical oxygen demand
BMP	Biochemical methane potential
COD	Chemical oxygen demand
CSWW	Cattle slaughterhouse wastewater
FOG	Fats, oil, and grease
HRT	Hydraulic retention time
OLR	Organic loading rat
SCOD	Soluble chemical oxygen demand
TSS	Total suspended solid
UASB	Upflow anaerobic sludge blanket
VSS	Volatile suspended solid
